# Exome-Wide Meta-Analysis Identifies Rare 3′-UTR Variant in ERCC1/CD3EAP Associated with Symptoms of Sleep Apnea

**DOI:** 10.3389/fgene.2017.00151

**Published:** 2017-10-18

**Authors:** Ashley van der Spek, Annemarie I. Luik, Desana Kocevska, Chunyu Liu, Rutger W. W. Brouwer, Jeroen G. J. van Rooij, Mirjam C. G. N. van den Hout, Robert Kraaij, Albert Hofman, André G. Uitterlinden, Wilfred F. J. van IJcken, Daniel J. Gottlieb, Henning Tiemeier, Cornelia M. van Duijn, Najaf Amin

**Affiliations:** ^1^Department of Epidemiology, Erasmus Medical Center, Rotterdam, Netherlands; ^2^Sleep and Circadian Neuroscience Institute, Nuffield Department of Clinical Neurosciences, University of Oxford, Oxford, United Kingdom; ^3^Department of Child and Adolescent Psychiatry, Erasmus Medical Center, Rotterdam, Netherlands; ^4^Framingham Heart Study, National Heart, Lung, and Blood Institute, Framingham, MA, United States; ^5^Population Sciences Branch, National Heart, Lung, and Blood Institute, Bethesda, MD, United States; ^6^Department of Biostatistics, School of Public Health, Boston University, Boston, MA, United States; ^7^Center for Biomics, Erasmus Medical Center, Rotterdam, Netherlands; ^8^Department of Internal Medicine, Erasmus Medical Center, Rotterdam, Netherlands; ^9^Netherlands Consortium for Healthy Ageing, Rotterdam, Netherlands; ^10^Department of Neurology, Erasmus Medical Center, Rotterdam, Netherlands; ^11^Department of Epidemiology, Harvard T. H. Chan School of Public Health, Boston, MA, United States; ^12^VA Boston Healthcare System, Boston, MA, United States; ^13^Departments of Medicine and Neurology, Brigham and Women's Hospital, Boston, MA, United States; ^14^Division of Sleep Medicine, Harvard Medical School, Boston, MA, United States; ^15^Department of Psychiatry, Erasmus Medical Center, Rotterdam, Netherlands

**Keywords:** sleep apnea syndromes, sleep, genetics, exome, sequence analysis, ERCC1, CD3EAP

## Abstract

Obstructive sleep apnea (OSA) is a common sleep breathing disorder associated with an increased risk of cardiovascular and cerebrovascular diseases and mortality. Although OSA is fairly heritable (~40%), there have been only few studies looking into the genetics of OSA. In the present study, we aimed to identify genetic variants associated with symptoms of sleep apnea by performing a whole-exome sequence meta-analysis of symptoms of sleep apnea in 1,475 individuals of European descent. We identified 17 rare genetic variants with at least suggestive evidence of significance. Replication in an independent dataset confirmed the association of a rare genetic variant (rs2229918; minor allele frequency = 0.3%) with symptoms of sleep apnea (*p*-value_meta_ = 6.98 × 10^−9^, β_meta_ = 0.99). Rs2229918 overlaps with the 3′ untranslated regions of *ERCC1* and *CD3EAP* genes on chromosome 19q13. Both genes are expressed in tissues in the neck area, such as the tongue, muscles, cartilage and the trachea. Further, *CD3EAP* is localized in the nucleus and mitochondria and involved in the tumor necrosis factor-alpha/nuclear factor kappa B signaling pathway. Our results and biological functions of *CD3EAP*/*ERCC1* genes suggest that the 19q13 locus is interesting for further OSA research.

## Introduction

Sleep is a complex and essential biological process that has been conserved across diverse animal species throughout evolution (Rechtschaffen, 1998). Although normal healthy sleep highly varies within and between adults (Van Dongen et al., 2005; Knutson et al., 2007; Mezick et al., 2009), it has to consist of adequate duration, good quality, proper timing and regularity, and the absence of sleep disturbances or disorders (Consensus Conference Panel et al., 2015). Several large epidemiological studies have shown that short or disturbed sleep is associated with various cognitive (Pilcher and Huffcutt, 1996; Yaffe et al., 2014), psychiatric (Lovato and Gradisar, 2014; Peters van Neijenhof et al., 2016; Cosgrave et al., in press) and health consequences e.g., diabetes mellitus (Gottlieb et al., 2005; Yaggi et al., 2006), activation of pro-inflammatory pathways (Patel et al., 2009), and cardiovascular diseases (Hoevenaar-Blom et al., 2011). One of the most common causes of short and disturbed sleep is sleep apnea.

Sleep apnea is a highly prevalent (Peppard et al., 2013) sleep breathing disorder, with obstructive sleep apnea (OSA) as the most common type (Mehra et al., 2007). OSA affects up to 38% of the general adult population (Senaratna et al., 2016) and untreated OSA has been associated with severe health problems (Young et al., 2002a) such as hypertension (Peppard et al., 2000; Pedrosa et al., 2011), cardiovascular disease (Shamsuzzaman et al., 2003; Marin et al., 2005; Gottlieb et al., 2010), stroke (Yaggi et al., 2005), type 2 diabetes (Shaw et al., 2008; Aurora and Punjabi, 2013; Kendzerska et al., 2014), impaired cognitive function (Kim et al., 1997; Yaffe et al., 2011), depression (Peppard et al., 2006), and increased mortality (Marshall et al., 2008; Young et al., 2008; Punjabi et al., 2009). The main characteristic of OSA is the partial or complete obstruction of the upper airways during sleep, causing oxyhemoglobin desaturations and arousals from sleep. This leads to sleep fragmentation and decreased periods of slow wave and REM sleep (McNicholas, 2008; American Academy of Sleep Medicine, 2014). Consequently, the two most common signs and symptoms of OSA are snoring and excessive daytime sleepiness (Gottlieb et al., 1999) where the latter can result in personal and occupational problems, and an increased risk of traffic and work-related accidents (Young et al., 2002a; McNicholas, 2008; American Academy of Sleep Medicine, 2014).

OSA is a complex trait influenced by both environment and genetics (Redline et al., 1995; Redline and Tishler, 2000) with obesity, age, and sex as most important risk factors (Redline et al., 1994; Bixler et al., 2001; Young et al., 2002a,b, 2004; Peppard et al., 2013). About 40% of the variance in apneic activity can be explained by genetic factors (Redline et al., 1995). At least half of the genetic contribution to sleep apnea acts through mechanisms independent of obesity (Patel et al., 2008). Previous genetic studies have focused on several candidate genes for breathing disorders, where the most studied genes are the angiotensin-converting enzyme gene (*ACE*) (Lin et al., 2004; Bostrom et al., 2007; Patel et al., 2007); apolipoprotein, allele E4 (*APOE* ϵ*4*) (Kadotani et al., 2001; Gottlieb et al., 2004); serotonin receptors and transporters genes (*5-HT2A, 5-HT2C, 5-HTT*) (Sakai et al., 2005; Ylmaz et al., 2005; Bayazit et al., 2006; Larkin et al., 2010; Qin et al., 2014); adrenergic receptors (*ADRB2/3*) (Mills et al., 1995; Grote et al., 2000); and tumor necrosis factor (*TNF*) (Riha et al., 2005; Popko et al., 2008; Bhushan et al., 2009). However, the results of these studies have been inconsistent or have yet to be confirmed (Sleiman and Hakonarson, 2011). Using linkage analysis, a method to identify the chromosomal location of the disease influencing genes, two regions on chromosome 2p16 and 19q13 were found to be suggestively linked with OSA independent of obesity (Palmer et al., 2003). Genome wide association studies (GWASs) could provide more information on common variants involved in the pathogenesis of OSA. Until now only a few GWASs have been reported for OSA. Loci in *GPR83* and *C6ORF183/CCDC162P* were found to be significantly associated with OSA (Cade et al., 2016), and a locus in the neuregulin-1 (*NRG1*) gene was suggestively implicated (Baik et al., 2015). Two other studies used customized or targeted genotyping arrays and identified loci in *PPARGC1B* (Kripke et al., 2015), *PTGER3* (Patel et al., 2012), *PLEK* (Patel et al., 2012), and *LPAR1* (Patel et al., 2012) to be associated with OSA. However, most of these findings were not replicated. Consequently, the genetic architecture of OSA remains largely unexplored.

In the present study we aimed to identify genetic variants associated with symptoms of sleep apnea, assessed using the Pittsburgh Sleep Quality Index (PSQI). We performed a GWAS using whole-exome sequence (WES) data of 1,475 individuals from two Dutch studies. Subsequently, we replicated our findings in an independent sample.

## Materials and methods

### Study populations

#### Discovery cohorts

The discovery sample consists of participants from two cohorts including the Erasmus Rucphen Family (ERF) study and the Rotterdam Study (RS) from The Netherlands.

ERF is a family-based study that includes inhabitants of a genetically isolated community in the Southwest of the Netherlands, ascertained as part of the Genetic Research in Isolated Population program. The ERF cohort includes ~3,000 living descendants of 22 founder couples, who had at least six children baptized in the community church. Individuals who were 18 years or older were invited to participate in the study. Data was collected between 2002 and 2005 (Pardo et al., 2005). The study was approved by the Medical Ethics Committee of the Erasmus Medical Center (EMC), Rotterdam, The Netherlands. All participants provided written informed consent and all investigations were carried out in accordance with the Declaration of Helsinki.

RS is a prospective cohort study ongoing since 1990, which aims to investigate determinants of disease occurrence and progression in the elderly (Hofman et al., 2015). Initially, the RS included 7,983 individuals of 55 years of age or over, living in the well-defined Ommoord district in Rotterdam, The Netherlands. All participants were examined at baseline by an at home interview and an extensive set of examinations in the research facility in Ommoord. The RS was approved by the Medical Ethics Committee of the EMC and by the Ministry of Health, Welfare and Sport of the Netherlands. All participants provided written informed consent to participate in the study. All investigations were carried out in accordance with the Declaration of Helsinki.

Study participants from ERF and RS were assessed for sleep phenotypes using a self-administered questionnaire including questions from the PSQI (Buysse et al., 1989). The PSQI has been specifically designed to measure sleep quality and sleep disturbances over a 1-month time interval. Symptoms of sleep apnea were assessed by asking the participants “How often did you or your partner notice long pauses between breaths while asleep?” Answers were provided on a categorical scale ranging from 1 to 4 (1. not during the past month; 2. less than once per week; 3. once or twice per week; 4. more than twice per week). Symptoms of sleep apnea were assessed in 1,366 ERF participants and 2,660 RS participants, where for the latter data of the fourth visit was used as it had the largest participation.

#### Replication cohort

The replication sample included participants from the offspring cohort of the population-based prospective Framingham Heart Study (FHS) (Dawber et al., 1951). The offspring cohort was recruited between 1971 and 1975, including 5,124 offspring of the original FHS cohort and their spouses (Kannel et al., 1979). The study was approved by the Institutional Review Board for Human Research of the Boston University Medical Center, Boston, MA, USA. Each participant provided written informed consent.

FHS has collected sleep data using the Sleep Heart Health Study sleep habits questionnaire (Quan et al., 1997). Symptoms of sleep apnea scores were constructed as a combination of the following questions: “A. Are there times when you stop breathing during your sleep?” with answers “yes”, ”no”, “I don't know” and “B. If yes to question A: How often do you have times when you stop breathing during your sleep?”. Answers to question B were provided on a categorical scale ranging from 1 to 5 (1. Rarely, less than one night per week; 2. Sometimes, one or two nights per week; 3. Frequently, three to five nights per week; 4. always or almost always, six or seven nights per week; 5. I don't know). Individuals with answers “I don't know” were excluded, since this option is not available in the PSQI. The constructed symptoms of sleep apnea score had answers ranging from 1 to 4, matching the PSQI: 1. not during the past month (A2); 2. less than once per week (A1 and B1); 3. once or twice per week (A1 and B2); 4. more than twice per week (A1 and B3 or A1 and B4).

### Sequencing and quality control

#### Discovery cohorts

In ERF Genomic DNA was extracted from peripheral venous blood utilizing the salting out method (Miller et al., 1988). Exomes of 1,336 ERF participants were sequenced at the Erasmus Center for Biomics of the Cell Biology department of the EMC, The Netherlands, using the Agilent V4 capture kit on an Illumina HiSeq2000 sequencing machine with the TruSeq Version 3 protocol (Amin et al., 2016b). The sequence reads were aligned to the human genome build 19 (hg19) using Burrows Wheeler Aligner (BWA) (Li and Durbin, 2009) and the NARWHAL pipeline (Brouwer et al., 2012). Aligned reads were further processed using IndelRealigner, MarkDuplicates and TableRecalibration tools from the Genome Analysis Toolkit (GATK) (Mckenna et al., 2010), and Picard (http://broadinstitute.github.io/picard/). Genetic variants were called using the GATK UnifiedGenotyper tool. Individuals with low concordance to genotyping array or with a low call rate and low quality variants (Phred quality score <30, call rate <90%) and out of Hardy-Weinberg equilibrium (HWE) (*p* < 10^−6^), were removed. The final dataset for ERF included 528,617 single nucleotide variants (SNVs) in 1,308 individuals (Amin et al., 2016b) of whom 654 individual also had phenotype data on symptoms of sleep apnea available.

Exomes of 2,628 individuals from the RS population were sequenced at the Human Genotyping facility of the Internal Medicine department at the EMC, the Netherlands, to an average depth of 54x using the Nimblegen SeqCap EZ V2 capture kit on an Illumina Hiseq2000 sequencer using the TruSeq Version 3 protocol (Amin et al., 2016b). The sequenced reads were aligned to hg19 using BWA (Li and Durbin, 2009). Subsequently, the aligned reads were processed further using Picard's MarkDuplicates, SAMtools (Li et al., 2009), and GATK (Mckenna et al., 2010). Genetic variants were called using the Haplotypecaller from GATK. Samples with low concordance to genotyping array (<95%), low transition to transversion ratio (<2.3) and high heterozygote to homozygote ratio (>2.0) were removed and additionally SNVs with a low call rate (<90%) and out of HWE (*p* < 10^−8^) were also removed from the data. The final dataset included 600,806 SNVs in 2,356 individuals (Amin et al., 2016a) of whom 821 individuals also had phenotype data on symptoms of sleep apnea available.

For both ERF and RS, file handling and formatting was done using VCFtools (Danecek et al., 2011) and PLINK (Purcell et al., 2007) (http://pngu.mgh.harvard.edu/purcell/plink/). Annotation of the variants was performed using SeattleSeq Annotation 138 (http://snp.gs.washington.edu/SeattleSeqAnnotation138/).

#### Replication cohort

In FHS exomes of 1,271 participants were sequenced using Illumina HiSeq2000 and 2500 platforms. DNA samples were constructed into Illumina paired-end pre-capture libraries according to the manufacturer's protocol. For exome capture, two, four or six pre-capture libraries were pooled together and hybridized to the HGSC VCRome 2.1 design (Bainbridge et al., 2011) (42 Mb, NimbleGen). After sequencing the HGSC Mercury analysis pipeline (https://www.hgsc.bcm.edu/content/mercury) and Illumina CASAVA software were used to perform sequencing analysis and to de-multiplex the pooled samples. Sequenced reads were aligned to Genome Reference Consortium Human Build 37 (GRCh37) using BWA (Li and Durbin, 2009) producing BAM files (Li et al., 2009). The aligned reads were recalibrated using GATK (Depristo et al., 2011) together with BAM sorting, duplicate read marking, and realignment near insertions or deletions. SNVs, insertions and deletions were called using Atlas2 (Challis et al., 2012). SNVs were excluded with low SNV posterior probability (<0.95), low variant read count (<3), variant read ratio <0.25 or >0.75, strand-bias of more than 99% variant reads in a single strand direction, or total coverage <10. Reference calls with <10 × coverage were also set to missing. Variants were excluded outside exon capture regions (VCRome 2.1), multi-allelic sites, monomorphic sites, missing rate >20%, mappability score <0.8, mean depth of coverage >500, or not fulfilling HWE (*p* < 5 × 10^−6^). Samples were excluded with missingness >20%, less than 6 SD from mean depth, more than 6 SD for singleton count, or outside of 6 SD for heterozygous to homozygous ratio or transition to transversion ratio. Variants were annotated using ANNOVAR (Wang et al., 2010) and dbNSFP v2.0 (https://sites.google.com/site/jpopgen/dbNSFP) according to the GRCh37 reference genome and National Center for Biotechnology Information RefSeq. The final dataset included 1,749,755 SNVs in 1,271 individuals of whom 472 individuals also had phenotype data on symptoms of sleep apnea available.

### Statistical analyses

Descriptive analysis was performed using IBM SPSS Statistics version 21 (IBM Corp. Released 2012. IBM SPSS Statistics for Windows, Version 21.0. Armonk, NY: IBM Corp.). Study specific exome analyses and meta-analysis of the individual study data were performed using the seqMeta v1.5 library of the R software (http://cran.r-project.org/web/packages/seqMeta/). Single variant association analysis was performed by assuming an additive effect. In ERF and FHS a linear mixed effects model was used adjusting for familial relationships by including the kinship matrix. To account for population stratification in the RS, we tested the association of ten principal components with the phenotype. None of them was significantly associated with symptoms of sleep apnea and we did not include them in the analysis. The regression analysis was performed using the four categories of symptoms of sleep apnea score as a continuous trait, adjusting for the three main risk factors for OSA; age, sex and body mass index (BMI) (kg/m^2^). Meta-analysis was performed using a fixed effects model. Variants that were present in both discovery cohorts (ERF and RS, 115,526 variants) were tested for association, giving a Bonferroni corrected *p*-value threshold of 4.3 × 10^−7^. All variants that showed significant or suggestive (*p* < 1.0 × 10^−6^) association signals in the discovery samples, were tested for replication in FHS. Bonferroni correction was also applied to correct for multiple testing in the replication stage.

## Results

Descriptive statistics of the study populations are presented in Table 1. The mean age in RS was 75 years (${\bar{x}}_{\text{BMI}}$ = 27.4 kg/m^2^), where the mean age in ERF was 46 years (${\bar{x}}_{\text{BMI}}$ = 26.7 kg/m^2^) and 59 years in FHS (${\bar{x}}_{\text{BMI}}$ = 27.5 kg/m^2^). The prevalence of symptoms of sleep apnea was higher in the ERF population, where 16.8% of the participants reported to have experienced apneas during the last month, compared to 11.6 and 6.6% of the RS and FHS participants, respectively (Table 2).

**Table 1:** Descriptive statistics of the study populations.

	**ERF**	**RS**	**FHS**
*N*	654	821	472
Age (years), mean ± SD	46.4 ± 13.4	75.0 ± 6.1	59.2 ± 9.4
Male (%)	42.5	46.8	48.5
BMI (kg/m^2^), mean ± SD	26.7 ± 4.4	27.4 ± 4.0	27.5 ± 4.7

*ERF, Erasmus Rucphen Family study; RS, Rotterdam Study; FHS, Framingham Heart Study; N, number of participants; BMI, body mass index*.

**Table 2:** Answers to the sleep apnea question for the discovery and replication populations.

		**ERF (%)**	**RS (%)**	**FHS (%)**
How often did you or your partner notice long pauses between breaths while asleep (a so-called sleep apnea)?	Not during the last month	544 (83.2)	726 (88.4)	441 (93.4)
	Less than once a week	48 (7.3)	44 (5.4)	16 (3.4)
	Once or twice a week	32 (4.9)	32 (3.9)	6 (1.3)
	More than twice a week	30 (4.6)	19 (2.3)	9 (1.9)
	Total	654	821	472

*ERF, Erasmus Rucphen Family study; RS, Rotterdam Study; FHS, Framingham Heart Study*.

**Figure 1:**
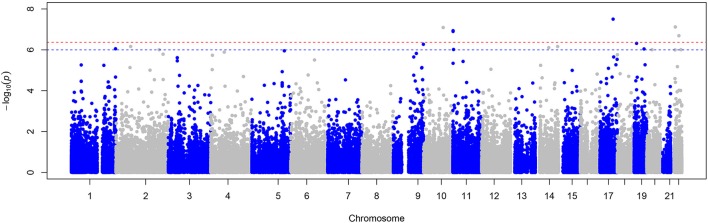
Manhattan plot of the meta-analysis of symptoms of sleep apnea. This plot shows −log_10_ transformed *p*-values (y-axis) for all SNPs present in the meta-analysis according to their position on each chromosome (x-axis). The red dashed line represents the Bonferroni corrected *p*-value threshold for significance (*p* < 4.3 × 10^−7^) and the blue dashed line indicates the threshold for suggestive associations (*p* < 1.0 × 10^−6^).

**Figure 2:**
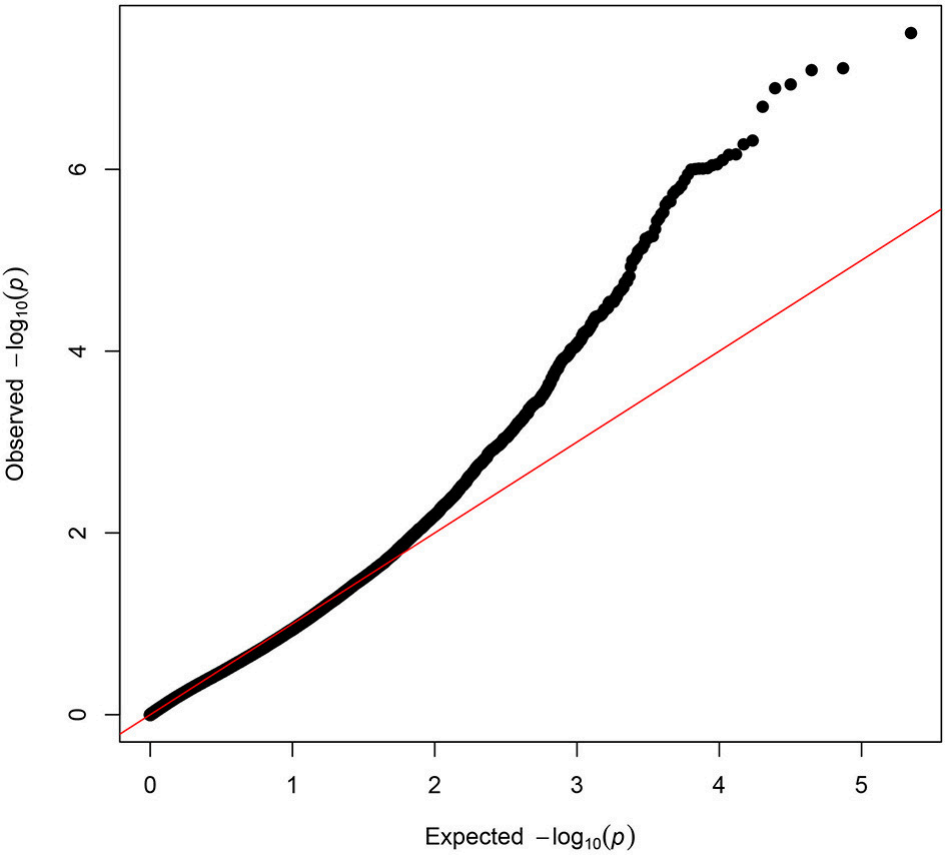
Quantile-Quantile plot of the meta-analysis of symptoms of sleep apnea. The QQ-plot shows the observed *p*-values plotted on the y-axis against the expected values of the test statistics on the x-axis (X^2^-distribution). The red line shows the distribution under the null hypothesis.

The exome-wide association results and the distribution of the test statistic (λ = 1.02) are illustrated in Figures 1, 2 respectively. Significant associations of symptoms of sleep apnea were observed with six rare variants [minor allele frequency (MAF) <1%] (located in *ACE, AIFM3, LIPJ, MUC2, AP2A2, SH3BP1)* (Table 3). Suggestive associations of symptoms of sleep apnea were observed with 11 rare variants (located in *KANK2, LCN6, TRAF3, PLEK, HIF1A, SLC45A3, ERCC1*/*CD3EAP, MRGPRE, GRAMD4, TYW5, CST5*) (Table 3). Of all 17 variants, only seven were polymorphic in the replication sample and could be tested for association (Table 4). Of the six significantly associated variants, two could be tested for association with symptoms of sleep apnea in the FHS (located in *MUC2* and *SH3BP1*).

**Table 3:** Meta-analysis association results, filtered on *p* < 1.0 × 10^−6^.

**Marker name**	**Gene**	**Chr**	**Position**	**Minor/major**	CADD^*^	Function GVS^*^	Poly Phen2^*^	GERP score^*^	**ERF (*N* = 654)**				**RS (*N* = 821)**				**Meta-analysis (*N* = 1,475)**			
									**MAF**	**Beta^†^**	**SE**	***p*-value**	**MAF**	**Beta^†^**	**SE**	***p*-value**	**MAF**	**Beta^†^**	**SE**	***p*-value**
rs137910205	*ACE*	17	61,561,775	A/G	0.50	Synonymous	–	−9.94	0.001	2.46	0.73	7.37 × 10^−04^	0.001	2.63	0.60	1.15 × 10^−05^	0.001	2.56	0.46	3.15 × 10^−08^
rs178276	*AIFM3*	22	21,331,950	C/G	6.73	Intron	–	−3.74	0.002	–0.29	0.52	5.75 × 10^−01^	0.004	1.53	0.25	5.03 × 10^−10^	0.003	1.19	0.22	7.68 × 10^−08^
rs77091298	*LIPJ*	10	90,356,568	G/T	15.40	Missense	0.60	4.12	0.002	1.89	0.52	2.74 × 10^−04^	0.001	1.68	0.42	7.61 × 10^−05^	0.001	1.76	0.33	8.04 × 10^−08^
rs9735156	*MUC2*	11	1,093,641	C/T	1.95	Synonymous	–	−2.97	0.001	2.46	0.73	7.78 × 10^−04^	0.001	1.77	0.42	2.93 × 10^−05^	0.001	1.94	0.37	1.16 × 10^−07^
11:977099	*AP2A2*	11	977,099	G/A	15.33	Missense	0.30	2.96	0.001	2.25	0.73	2.14 × 10^−03^	0.001	2.59	0.60	1.61 × 10-^05^	0.001	2.45	0.46	1.27 × 10^−07^
rs149928566	*SH3BP1*	22	38,039,746	T/C	13.28	Missense	0.72	−2.34	0.002	0.03	0.53	9.52 × 10^−01^	0.005	1.11	0.20	3.14 × 10^−08^	0.004	0.97	0.19	2.04 × 10^−07^
rs117057052	*KANK2*	19	11,277,278	C/T	3.31	Missense	0.37	3.11	0.001	2.39	0.73	1.09 × 10^−03^	0.001	1.67	0.42	8.60 × 10^−05^	0.001	1.85	0.37	4.78 × 10^−07^
9:139642861	*LCN6*	9	139,642,861	C/T	2.75	Non-coding exon	–	−2.03	0.005	1.06	0.30	4.81 × 10^−04^	0.001	2.54	0.60	2.47 × 10^−05^	0.002	1.36	0.27	5.31 × 10^−07^
rs148461790	*TRAF3*	14	103,369,593	A/G	15.27	Missense near splice	0.43	4.53	0.001	1.66	0.73	2.28 × 10^−02^	0.001	2.73	0.60	5.23 × 10^−06^	0.001	2.30	0.46	6.83 × 10^−07^
rs34515106	*PLEK*	2	68,607,978	C/A	14.50	Missense	0.80	5.80	0.002	0.94	0.52	7.27 × 10^−02^	0.001	2.10	0.42	7.80 × 10^−07^	0.001	1.64	0.33	6.87 × 10^−07^
rs149348765	*HIF1A*	14	62,204,819	T/G	14.84	Missense	0.59	5.41	0.008	0.89	0.24	1.46 × 10^−04^	0.001	1.41	0.42	8.62 × 10^−04^	0.004	1.02	0.21	7.89 × 10^−07^
rs139592793	*SLC45A3*	1	205,632,166	T/C	6.52	Synonymous	–	1.32	0.002	1.22	0.43	4.24 × 10^−03^	0.001	2.67	0.60	8.42 × 10^−06^	0.001	1.71	0.35	8.80 × 10^−07^
rs2229918	*ERCC1, CD3EAP*	19	45,912,924	G/C	7.63	3-prime-UTR	–	0.57	0.003	0.49	0.37	1.80 × 10^−01^	0.003	1.37	0.27	3.39 × 10^−07^	0.003	1.07	0.22	8.98 × 10^−07^
rs191846883	*MRGPRE*	11	3,249,162	A/G	5.89	Synonymous	–	3.55	0.002	1.61	0.52	1.85 × 10^−03^	0.004	0.85	0.21	5.87 × 10^−05^	0.003	0.96	0.20	9.61 × 10^−07^
22:47058906	*GRAMD4*	22	47,058,906	T/C	1.44	Intron	–	−5.20	0.002	1.33	0.52	1.05 × 10^−02^	0.001	2.71	0.60	6.16 × 10^−06^	0.001	1.92	0.39	9.78 × 10^−07^
2:200803697	*TYW5*	2	200,803,697	A/G	38.00	Stop-gained	–	4.45	0.001	1.71	0.73	1.94 × 10-^02^	0.001	2.64	0.60	1.02 × 10^−05^	0.001	2.27	0.46	9.83 × 10^−07^
rs142729279	*CST5*	20	23,858,232	A/G	1.78	Synonymous	–	0.46	0.001	1.71	0.73	1.94 × 10^−02^	0.001	2.64	0.60	1.02 × 10^−05^	0.001	2.27	0.46	9.89 × 10^−07^

*Chr, Chromosome; CADD, Combined Annotation Dependent Depletion; GERP, Genomic Evolutionary Rate Profiling; ERF, Erasmus Rucphen Family study; RS, Rotterdam Study; MAF, Minor Allele Frequency, SE, Standard Error, –, unknown;*

* *SeattleSeq Annotation Database 138*.

† *All effects are reported for the minor allele*.

**Table 4:** Replication results, filtered on *p* < 1.0 × 10^−6^.

**Marker name**	**Gene**	**Meta-analysis (*N* = 1475)**				**Replication FHS (*N* = 472)**				**Meta-analysis (discovery and replication, *N* = 1947)**			
		**MAF**	**Beta^†^**	**SE**	***p*-value**	**MAF**	**Beta^†^**	**SE**	***p*-value**	**MAF**	**Beta^†^**	**SE**	***p*-value**
rs137910205	*ACE*	0.001	2.56	0.46	3.15 × 10^−08^	–	–	–	–	–	–	–	–
rs77091298	*LIPJ*	0.001	1.76	0.33	8.04 × 10^−08^	–	–	–	–	–	–	–	–
rs9735156	*MUC2*	0.001	1.94	0.37	1.16 × 10^−07^	0.002	−0.14	0.34	0.69	0.001	0.83	0.25	9.09 × 10^−04^
11:977099	*AP2A2*	0.001	2.45	0.46	1.27 × 10^−07^	–	–	–	–	–	–	–	–
rs149928566	*SH3BP1*	0.004	0.97	0.19	2.04 × 10^−07^	0.010	−0.16	0.16	0.33	0.005	0.33	0.12	7.69 × 10^−03^
rs117057052	*KANK2*	0.001	1.85	0.37	4.78 × 10^−07^	0.005	−0.12	0.22	0.59	0.002	0.39	0.19	3.66 × 10^−02^
9:139642861	*LCN6*	0.002	1.36	0.27	5.31 × 10^−07^	–	–	–	–	–	–	–	–
rs148461790	*TRAF3*	0.001	2.30	0.46	6.83 × 10^−07^	–	–	–	–	–	–	–	–
rs34515106	*PLEK*	0.001	1.64	0.33	6.87 × 10^−07^	–	–	–	–	–	–	–	–
rs149348765	*HIF1A*	0.004	1.02	0.21	7.89 × 10^−07^	0.002	–0.15	0.34	0.66	0.004	0.71	0.18	6.09 × 10^−05^
rs139592793	*SLC45A3*	0.001	1.71	0.35	8.80 × 10^−07^	0.002	–0.27	0.34	0.43	0.002	0.70	0.24	3.91 × 10^−03^
rs2229918	*ERCC1, CD3EAP*	0.003	1.07	0.22	8.98 × 10^−07^	0.003	0.87	0.28	1.84 × 10^−03^	0.003	0.99	0.17	6.98 × 10^−09^
rs191846883	*MRGPRE*	0.003	0.96	0.20	9.61 × 10^−07^	–	–	–	–	–	–	–	–
22:47058906	*GRAMD4*	0.001	1.92	0.39	9.78 × 10^−07^	–	–	–	–	–	–	–	–
2:200803697	*TYW5*	0.001	2.27	0.46	9.83 × 10^−07^	–	–	–	–	–	–	–	–
rs142729279	*CST5*	0.001	2.27	0.46	9.89 × 10^−07^	0.002	−0.02	0.34	0.95	0.001	0.78	0.28	4.35 × 10^−03^

*MAF, Minor Allele Frequency; SE, Standard Error; –, Not available; FHS, Framingham Heart Study*.

† *All effects are reported for the minor allele*.

**Figure 3:**
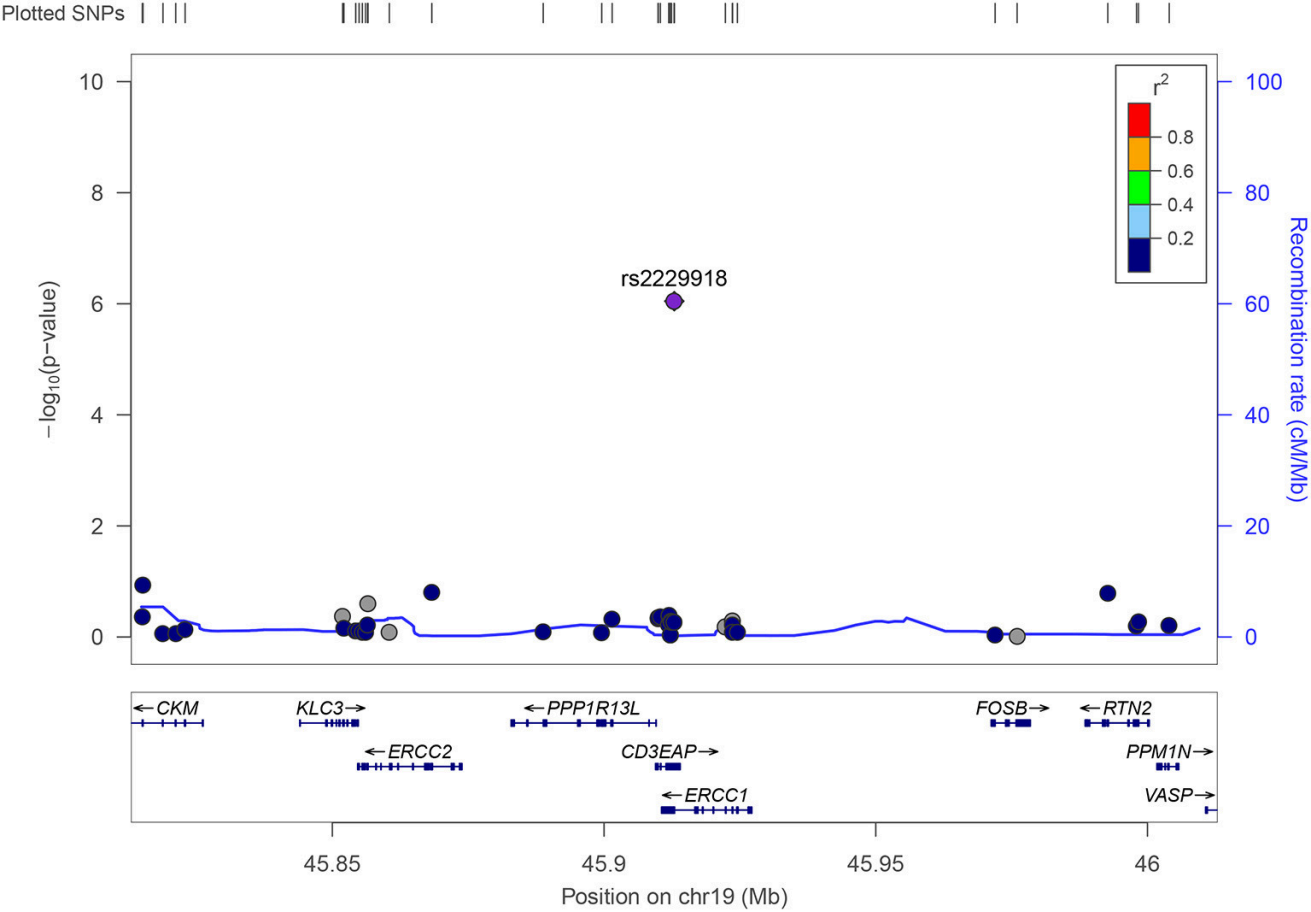
Regional association plot for rs2229918. Rs2229918 is located in purple. The dots show the variants tested in this region on chromosome 19. The −log10 transformed *p*-values are plotted on the y-axis and the genes and positions of the variants (Mb) in this region are depicted on the x-axis.

A significant association of symptoms of sleep apnea with rs2229918, located on chromosome 19q13 in the overlapping 3′-untranslated region (UTR) of the *ERRC1* and *CD3EAP* genes (Figure 3), was observed in the replication sample (*p* = 1.84 × 10^−3^). Moreover, both the frequency (MAF_FHS_ = 0.3%) and the effect size of the minor allele (G; β_FHS_ = 0.87) were consistent with that of the discovery cohorts (MAF = 0.3%, β = 1.07) suggesting that each copy of the minor allele (G) can result in a shift to a higher category in self-reported apnea symptoms (PSQI). Meta-analysing the discovery and replication cohorts yielded an increased significance of the association of rs2229918 with symptoms of sleep apnea (*p* = 6.98 × 10^−9^, β = 0.99).

## Discussion

This study aimed at identifying genetic variants associated with symptoms of sleep apnea by performing a meta-analysis of WES data. We identified a rare genetic variant (MAF = 0.3%), rs2229918, located in the shared 3′-UTR region of the *ERCC1* and *CD3EAP* genes with a large effect on symptoms of sleep apnea. We show significant replication of rs2229918 in an independent sample.

The *CD3EAP* gene is located in antisense orientation to *ERCC1* where the 3′-UTRs of both genes overlap. This type of 3′-UTR overlap is conserved in mice and yeast suggesting an important biological function (OMIM #107325). 3′-UTRs can be highly enriched for regulatory elements such as binding sites for regulatory proteins and microRNAs and therefore are most likely involved in post-transcriptional regulation (Xie et al., 2005). *ERCC1* encodes Excision Repair Cross-Complementation Group 1, a protein functioning in the nucleotide excision repair pathway and needed for the repair of DNA lesions but also involved in recombinational DNA repair and the repair of inter-strand crosslinks (Stelzer et al., 2016). Mutations in *ERCC1* have been linked to cerebro-oculo-facio-skeletal syndrome 4, a severe autosomal recessive disorder characterized by growth retardation, dysmorphic facial features, arthrogryposis, and neurologic abnormalities (OMIM #610758). *CD3EAP* is a component of RNA polymerase I which synthesizes ribosomal RNA precursors and is involved in poly(A) RNA binding and DNA-directed RNA polymerase activity (Stelzer et al., 2016). *CD3EAP* is localized in the nucleus and mitochondria and has two isoforms, isoform 1 is involved in UBTF-activated (Upstream Binding Transcription Factor, RNA Polymerase 1) transcription, while isoform 2 is a component of preformed T-cell receptor complex. *CD3EAP* is involved in multiple pathways including rRNA expression and RNA Polymerase 1 transcription related pathways; RNA polymerase I promotor escape and transcription; gene expression; and the TNF-alpha/NF-kB signaling pathway (Stelzer et al., 2016). Previous genetic studies have associated NF-kB-dependent genes, especially *TNF*-α, with OSA (Riha et al., 2005; Ryan et al., 2006; Popko et al., 2008; Bhushan et al., 2009). Moreover, NF-kB is thought to play a key role in mediation of the inflammatory and cardiovascular consequences of OSA (Ryan et al., 2005; Garvey et al., 2009). GeneNetwork (Fehrmann et al., 2015) (http://129.125.135.180:8080/GeneNetwork/) shows that both *ERCC1* and *CD3EAP* are expressed in tissues that may be related to obstruction of the upper airway or diseases of tissues/organs associated with OSA, such as muscle cells, cartilage, trachea, salivary glands, heart and heart ventricles, glucagon secreting cells, the neck and the tongue. This further supports that *ERCC1* and *CD3EAP* are interesting candidate genes for symptoms of sleep apnea.

Rs2229918 is located on chromosome 19q13, a previously identified region with suggestive evidence for linkage to OSA in European-Americans, independently of BMI (Palmer et al., 2003). Although *APOE*, a known candidate gene for OSA, is also located in this region, it did not show association with OSA in the present study. A previous study fine-mapped the *APOE* region and concluded that *APOE* does not explain the linkage signal, suggesting that *APOE* is not the causative locus (Larkin et al., 2006). Although the linkage analysis performed by Palmer et al. (2003) was redone by adding additional family members and families, the chromosome 19 region was not confirmed. However, this could be due to the genetic or disease heterogeneity (Larkin et al., 2008).

Additionally, there were six rare variants (MAF < 0.4%) that surpassed the Bonferroni corrected *p*-value threshold, of which three (located in *ACE, LIPJ* and *AP2A2*) were monomorphic in the FHS and could not be tested for replication. Our top finding, rs137910205, a synonymous variant, is located in the *ACE* (angiotensin converting enzyme) gene, one of the most studied genes for OSA. Previous studies found an association between the *ACE* insertion/deletion polymorphism and an increased risk of hypertension in OSA patients (Lin et al., 2004; Bostrom et al., 2007), although results are conflicting (Patel et al., 2007). Further, plasma activity of ACE has been found to be increased in untreated OSA patients (Barcelo et al., 2001). Both carriers of rs137910205 (1 in each cohort) reported the highest score for symptoms of sleep apnea, i.e. these individuals have experienced pauses in breathing at least twice per week. The second variant is the missense variant, rs77091298, located in the *LIPJ* (Lipase Family Member J) gene. GeneNetwork showed that *LIPJ* is expressed in the nasopharynx, neck, and muscle cells, all highly relevant tissues in the pathogenesis of OSA (Fehrmann et al., 2015). The third variant that could not be tested for replication, 11:977099, has not been identified before. The variant is located in the *AP2A2* gene (Adaptor Related Protein Complex 2 Alpha 2 Subunit), which is related to lipid binding (Stelzer et al., 2016). However, we caution against the interpretation of statistics when the number of carriers of the genetic variants is less than five. Larger sample sizes are needed to further investigate the possible association of these rare genetic variants with OSA.

This study has some limitations regarding the study design. We have used questionnaire data for the assessment of symptoms of sleep apnea, which could introduce bias (Fedson et al., 2012). Although reports of breathing pauses more than twice per week are highly predictive of polysomnographic sleep apnea, self- or partner-reported breathing pauses have low sensitivity (Young et al., 2002b). Individuals with sleep apnea who experience predominantly hypopneas (shallow breathing) rather than apneas may be less likely to be identified with questionnaire data, as these individuals and their partners may be less likely to recognize these events. Another limitation of using questionnaire data is that the discrimination between OSA, central sleep apnea and mixed sleep apnea is not possible. Although the prevalence of central sleep apnea is generally much lower than OSA in particular in general population samples (Donovan and Kapur, 2016). Another limitation is that our findings might not be generalizable to other populations as all studies used in this analysis are predominantly European or European American populations. Previous studies have shown a difference in prevalence of sleep apnea between populations, where young African Americans may be at increased risk for sleep apnea (Redline et al., 1997) and had a higher apnea-hypopnea index relative to European Americans with OSA/hypopnea syndrome (Pranathiageswaran et al., 2013). The frequency of the rs2229918 minor allele (G), based on the 1000 Genomes data, also differs across populations (https://www.ncbi.nlm.nih.gov/variation/tools/1000genomes/). Lastly, sleep apnea is a complex and heterogeneous disease influenced by many risk factors such as obesity, age, gender (Redline et al., 1994; Bixler et al., 2001; Young et al., 2002b,a, 2004; Peppard et al., 2013), craniofacial and upper airway abnormalities (Mayer et al., 1996; White, 2005), race (Redline et al., 1997; Li et al., 2000), alcohol intake (Young et al., 2002a), smoking (Wetter et al., 1994), and reduced nasal patency due to congestion and respiratory allergies (Young et al., 1997). Despite this phenotypic complexity, we have identified and replicated a rare variant associated with symptoms of sleep apnea. However, we have only used one replication sample and additional studies should further investigate the association of rs2229918 with sleep apnea using objective measurements.

To conclude, this first meta-analysis of symptoms of sleep apnea using WES data identified a rare genetic variant, rs2229918 (MAF 0.3%), located in the 3′-UTR of *ERCC1* and *CD3EAP*, associated with symptoms of sleep apnea. Both genes are interesting candidate genes for (symptoms of) sleep apnea based on their function and expression in tissues relevant for the pathogenesis of the disease. However, the involvement of rs2229918 in OSA pathology should be further examined in larger datasets with more objective measurements.

## Author contributions

AvdS, CvD, and NA contributed to the conceptualization and design of this work; AvdS and CL were involved in the analysis of the data; AvdS, AL, DK, DG, HT, CvD, and NA were involved in interpretation of the results; AvdS and NA were involved in writing and revising the manuscript; AL, DK, RB, JvR, MvdH, RK, AH, AU, WvI, HT, and CvD were involved in data collection/preparation; AL, DK, CL, RB, JvR, MvdH, RK, AH, AU, WvI, DG, HT, and CvD contributed to the interpretation of the data, read and approved the final manuscript.

### Conflict of interest statement

NA reports grants from Netherlands Brain Foundation, outside the submitted work. DG reports grants from NIH, during the conduct of the study; personal fees from VIVUS, Inc., outside the submitted work. RK reports grants from Netherlands Genomics Initiative (NGI), grants from Biobanking and Biomolecular Research Infrastructure Netherlands (BBMRI-NL), during the conduct of the study. AL reports grants and non-financial support from Big Health Ltd., outside the submitted work. HT reports grants from Netherlands Organization for Health Research and Development, during the conduct of the study. The other authors declare that the research was conducted in the absence of any commercial or financial relationships that could be construed as a potential conflict of interest.

## Abbreviations

BMI: body mass index
BWA: Burrows Wheeler Aligner
EMC: Erasmus Medical Center
ERF: Erasmus Rucphen Family
FHS: Framingham Heart Study
GATK: Genome Analysis Toolkit
GRCh37: Genome Reference Consortium Human Build 37
GWAS: genome wide association study
Hg19: human genome build 19
HWE: Hardy-Weinberg equilibrium
MAF: minor allele frequency
OSA: obstructive sleep apnea
PSQI: Pittsburgh Sleep Quality Index
RS: Rotterdam Study
SNV: single nucleotide variant
UBTF: upstream binding transcription factor
UTR: untranslated region
WES: whole-exome sequence.
